# 3-(4-Meth­oxy­phen­yl)-1,3-selenazolo[2,3-*b*][1,3]benzo­thia­zol-4-ium hydrogen sulfate

**DOI:** 10.1107/S1600536813009288

**Published:** 2013-04-13

**Authors:** Gunay Z. Mammadova, Zhanna V. Matsulevich, Galina N. Borisova, Alexander V. Borisov, Victor N. Khrustalev

**Affiliations:** a Baku State University, Z. Khalilov St 23, Baku, AZ-1148, Azerbaijan; b R.E. Alekseev Nizhny Novgorod State Technical University, 24 Minin St, Nizhny Novgorod, 603950, Russian Federation; c X-Ray Structural Centre, A.N. Nesmeyanov Institute of Organoelement Compounds, Russian Academy of Sciences, 28 Vavilov St, B-334, Moscow 119991, Russian Federation

## Abstract

The title compound, C_16_H_12_NOSSe^+^·HSO_4_^−^, was obtained from a mixture of 3-(4-meth­oxy­phen­yl)[1,3]selenazolo[2,3-*b*][1,3]benzo­thia­zol-4-ium chloride and potassium hydrogen sulfate. In the cation, the benzene ring is twisted by 71.62 (7)° from the tricycle mean plane. In the crystal, O—H⋯O hydrogen bonds link the anions into chains along [100]. The anions in adjacent chains are linked *via* weak C—H⋯O hydrogen bonds. The crystal packing exhibits short inter­molecular contacts between the chalcogen unit and the O atoms: Se⋯O(anion) 2.713 (3), Se⋯O(cation) 2.987 (3) and S⋯O(anion) 2.958 (3) Å.

## Related literature

For details of the synthesis and the biological properties of selenium- and nitro­gen-containing heterocycles, see: Back (2009 ▶); Mlochowski & Giurg (2009 ▶); Mukherjee *et al.* (2010 ▶); Selvakumar *et al.* (2010 ▶). For the synthesis of the starting compound, 3-(4-meth­oxy­phen­yl)[1,3]selenazolo[2,3-*b*][1,3]benzo­thia­zol-4-ium chloride, see: Borisov *et al.* (2012 ▶).
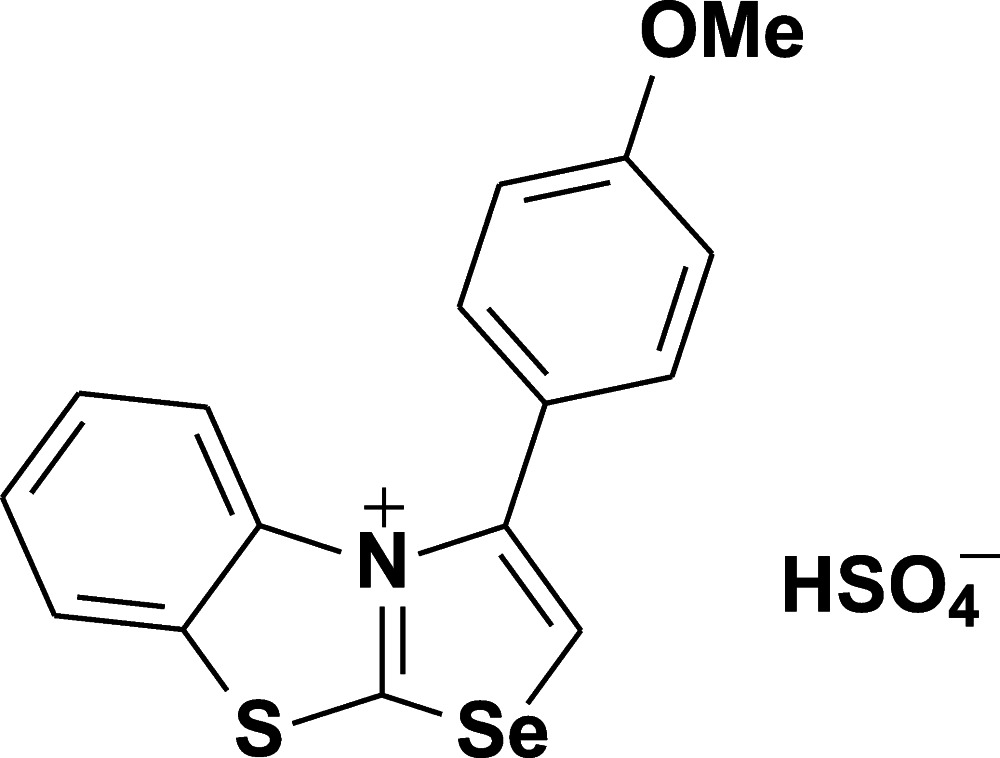


## Experimental

### 

#### Crystal data

C_16_H_12_NOSSe^+^·HO_4_S^−^
*M* *_r_* = 442.35
Monoclinic, 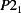

*a* = 4.6408 (8) Å
*b* = 18.263 (3) Å
*c* = 9.4482 (16) Å
β = 94.294 (3)°
*V* = 798.6 (2) Å^3^
*Z* = 2
Mo *K*α radiation
μ = 2.64 mm^−1^
*T* = 100 K
0.30 × 0.18 × 0.02 mm

#### Data collection

Bruker SMART 1K CCD diffractometer
Absorption correction: multi-scan (*SADABS*; Sheldrick, 1998 ▶) *T*_min_ = 0.505, *T*_max_ = 0.949
8638 measured reflections
4145 independent reflections
3555 reflections with *I* > 2σ(*I*)
*R*_int_ = 0.052

#### Refinement

*R*[*F* ^2^ > 2σ(*F* ^2^)] = 0.040
*wR*(*F* ^2^) = 0.085
*S* = 0.97
4145 reflections
227 parameters
1 restraint
H-atom parameters constrained
Δρ_max_ = 0.74 e Å^−3^
Δρ_min_ = −0.99 e Å^−3^
Absolute structure: Flack (1983 ▶), 2075 Friedel pairs
Flack parameter: 0.038 (9)

### 

Data collection: *SMART* (Bruker, 1998 ▶); cell refinement: *SAINT* (Bruker, 1998 ▶); data reduction: *SAINT*; program(s) used to solve structure: *SHELXTL* (Sheldrick, 2008 ▶); program(s) used to refine structure: *SHELXTL*; molecular graphics: *SHELXTL*; software used to prepare material for publication: *SHELXTL*.

## Supplementary Material

Crystal structure: contains datablock(s) global, I. DOI: 10.1107/S1600536813009288/cv5401sup1.cif

Structure factors: contains datablock(s) I. DOI: 10.1107/S1600536813009288/cv5401Isup2.hkl

Supplementary material file. DOI: 10.1107/S1600536813009288/cv5401Isup3.cml

Additional supplementary materials:  crystallographic information; 3D view; checkCIF report

## Figures and Tables

**Table 1:** Hydrogen-bond geometry (Å, °)

*D*—H⋯*A*	*D*—H	H⋯*A*	*D*⋯*A*	*D*—H⋯*A*
O5—H5*O*⋯O4^i^	0.91	1.80	2.610 (4)	147
C6—H6⋯O4^ii^	0.95	2.54	3.494 (5)	178
C8—H8⋯O2^iii^	0.95	2.26	3.022 (5)	137

Symmetry codes: (i) 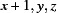; (ii) 
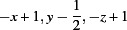
; (iii) 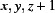.

**Fig. 1.**
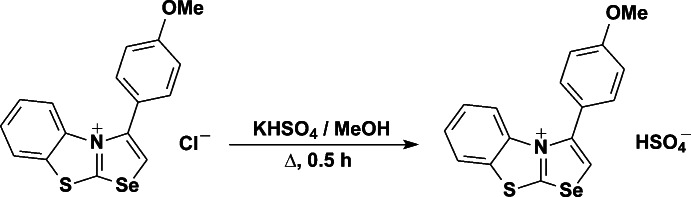
Reaction of 3-(4-methoxyphenyl)[1,3]selenazolo[2,3-b][1,3]benzothiazol-4-ium chloride with potassium hydrogen sulfate.

**Fig. 2.**
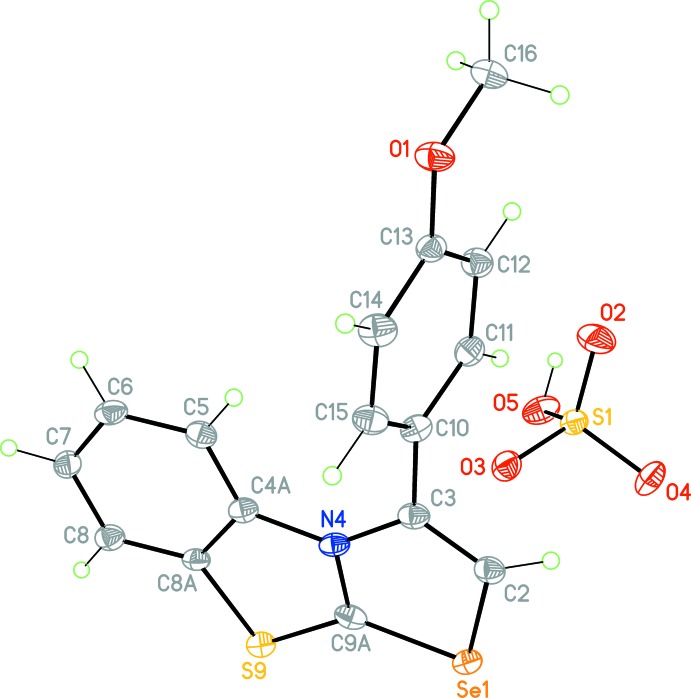
Molecular structure of I. Displacement ellipsoids are shown at the 50% probability level. H atoms are presented as small spheres of arbitrary radius.

**Fig. 3.**
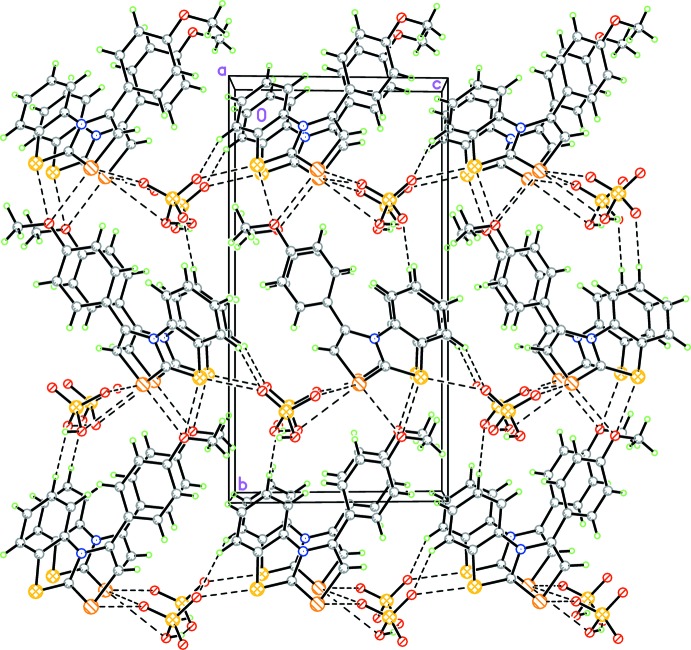
A portion of crystal packing of I viewed approximately down the a axis. Dashed lines indicate the intermolecular O—H···O and C—H···O hydrogen bonds as well as the non-valent Se···O and S···O attractive interactions.

## supplementary crystallographic information

### Comment

In the last years, the selenium- and nitrogen-containing heterocycles have attracted considerable attention owing to the variety of their pharmacological properties (Back, 2009; Mlochowski & Giurg, 2009; Mukherjee *et al.*, 2010; Selvakumar *et al.*, 2010). This article describes the structure of 3-(4-methoxyphenyl)[1,3]selenazolo[2,3-*b*][1,3]benzothiazol-4-ium hydrogen sulfate, [C_16_H_12_NOSSe]^+^.[HSO_4_]^-^, (I), which was obtained by the reaction of 3-(4-methoxyphenyl)[1,3]selenazolo[2,3-*b*][1,3]benzothiazol-4-ium chloride (Borisov *et al.*, 2012) with potassium hydrogen sulfate (Figure 1).

The compound I is a salt consisting of 3-(4-methoxyphenyl)[1,3]selenazolo[2,3-*b*][1,3]benzothiazol-4-ium cation and hydrogen sulfate anion (Figure 2). The cation of I comprises a fused tricyclic system containing two five-membered rings (1,3-selenazole and 1,3-thiazole) and one six-membered ring (benzene). The tricyclic system is practically planar (r.m.s deviation = 0.020 Å). The methoxy group is almost coplanar to the plane of the phenyl substituent (the C12—C13—O1—C16 torsion angle is 1.7 (5)°). The dihedral angle between the mean planes of the tricyclic system and methoxyphenyl fragment is 71.82 (6)°.

In the crystal, anions form chains along the *a* axis through the intermolecular O—H···O hydrogen bonding interactions (Table 1, Figure 3). Weak intermolecular C—H···O hydrogen bonds (Table 1, Figure 3) as well as non-valent attractive Se···O (Se1···O3^b^ 2.713 (3), Se1···O1^c^ 2.987 (3), Se1···O3 3.366 (3), Se1···O5^b^ 3.423 (3) Å) and S···O [S9···O2^a^ 2.958 (3) and S9···O1^c^ 3.050 (3) Å] interactions consolidate further the three-dimensional-crystal packing (Figure 3) [symmetry codes: (a) *x*, *y*, *z* + 1; (b) *x*–1, *y*, *z*; (c) –*x*, *y* + 0.5, –*z* + 1].

### Experimental

A mixture of 3-(4-methoxyphenyl)[1,3]selenazolo[2,3-*b*][1,3]benzothiazol-4-ium chloride (0.19 g, 0.5 mmol) with KHSO_4_ (0.072 g, 0.53 mmol) in CH_3_OH (20 ml) was refluxed for 0.5 h to dissolve the starting materials. After that the reaction mixture was concentrated in *vacuo*. Then CH_2_Cl_2_ (20 ml) was added to the solid to give precipitate of KCl, which was separated by filtration. The filtrate was concentrated in *vacuo*. The solid was re-crystallized from CH_2_Cl_2_ to give I as colorless crystals. Yield is 93%. *M*.p. = 510–512 K. ^1^H NMR (DMSO-*d*_6_, 600 MHz, 302 K): δ = 8.42 (1*H*, d, *J* = 8.0, H8), 8.23 (1*H*, s, H2), 7.63 (1*H*, t, *J* = 8.0, H7), 7.59 (2*H*, d, *J* = 8.8, H11, H15), 7.48 (1*H*, t, *J* = 8.0, H6), 7.25 (2*H*, d, *J* = 8.8, H12, H14), 6.81 (1*H*, d, *J* = 8.1, H5), 3.87 (3*H*, s, O*Me*). Anal. Calcd. for C_16_H_13_NO_5_S_2_Se: C, 43.45; H, 2.96; N, 3.17. Found: C, 43.37; H, 2.93; N, 3.12.

### Refinement

The hydroxyl hydrogen atom was localized in the difference-Fourier map and included in the refinement with fixed positional and isotropic displacement parameters [*U*_iso_(H) = 1.5*U*_eq_(O)]. The other hydrogen atoms were placed in calculated positions with C—H = 0.95 Å (CH) and 0.98 Å (CH_3_) and refined in the riding model with fixed isotropic displacement parameters [*U*_iso_(H) = 1.5*U*_eq_(C) for the CH_3_ group and 1.2*U*_eq_(C) for the CH groups].

### Crystal data

**Table:**

C_16_H_12_NOSSe^+^·HO_4_S^−^	*F*(000) = 444
*M**_r_* = 442.35	*D*_x_ = 1.840 Mg m^−^^3^
Monoclinic, *P*2_1_	Mo *K*α radiation, λ = 0.71073 Å
Hall symbol: P 2yb	Cell parameters from 2780 reflections
*a* = 4.6408 (8) Å	θ = 2.2–28.3°
*b* = 18.263 (3) Å	µ = 2.64 mm^−^^1^
*c* = 9.4482 (16) Å	*T* = 100 K
β = 94.294 (3)°	Plate, colourless
*V* = 798.6 (2) Å^3^	0.30 × 0.18 × 0.02 mm
*Z* = 2	

### Data collection

**Table:**

Bruker SMART 1K CCD diffractometer	4145 independent reflections
Radiation source: fine-focus sealed tube	3555 reflections with *I* > 2σ(*I*)
Graphite monochromator	*R*_int_ = 0.052
φ and ω scans	θ_max_ = 29.0°, θ_min_ = 2.2°
Absorption correction: multi-scan (*SADABS*; Sheldrick, 1998)	*h* = −6→6
*T*_min_ = 0.505, *T*_max_ = 0.949	*k* = −24→24
8638 measured reflections	*l* = −12→12

### Refinement

**Table:**

Refinement on *F*^2^	Secondary atom site location: difference Fourier map
Least-squares matrix: full	Hydrogen site location: difference Fourier map
*R*[*F*^2^ > 2σ(*F*^2^)] = 0.040	H-atom parameters constrained
*wR*(*F*^2^) = 0.085	*w* = 1/[σ^2^(*F*_o_^2^) + (0.001*P*)^2^] where *P* = (*F*_o_^2^ + 2*F*_c_^2^)/3
*S* = 0.97	(Δ/σ)_max_ < 0.001
4145 reflections	Δρ_max_ = 0.74 e Å^−^^3^
227 parameters	Δρ_min_ = −0.99 e Å^−^^3^
1 restraint	Absolute structure: Flack (1983), 2075 Friedel pairs
Primary atom site location: structure-invariant direct methods	Flack parameter: 0.038 (9)

### Special details

**Table:**

Geometry. All e.s.d.'s (except the e.s.d. in the dihedral angle between two l.s. planes) are estimated using the full covariance matrix. The cell e.s.d.'s are taken into account individually in the estimation of e.s.d.'s in distances, angles and torsion angles; correlations between e.s.d.'s in cell parameters are only used when they are defined by crystal symmetry. An approximate (isotropic) treatment of cell e.s.d.'s is used for estimating e.s.d.'s involving l.s. planes.
Refinement. Refinement of *F*^2^ against ALL reflections. The weighted *R*-factor *wR* and goodness of fit *S* are based on *F*^2^, conventional *R*-factors *R* are based on *F*, with *F* set to zero for negative *F*^2^. The threshold expression of *F*^2^ > σ(*F*^2^) is used only for calculating *R*-factors(gt) *etc*. and is not relevant to the choice of reflections for refinement. *R*-factors based on *F*^2^ are statistically about twice as large as those based on *F*, and *R*- factors based on ALL data will be even larger.

### Fractional atomic coordinates and isotropic or equivalent isotropic displacement parameters (Å^2^)

**Table:**

	*x*	*y*	*z*	*U*_iso_*/*U*_eq_	
Se1	−0.35253 (8)	0.72534 (2)	0.59512 (4)	0.01626 (9)	
C2	−0.3094 (9)	0.6448 (2)	0.4783 (4)	0.0173 (8)	
H2	−0.4181	0.6389	0.3898	0.021*	
C3	−0.1125 (9)	0.5955 (2)	0.5288 (4)	0.0165 (8)	
N4	0.0200 (7)	0.61551 (17)	0.6630 (4)	0.0147 (7)	
C4A	0.2352 (8)	0.5819 (2)	0.7546 (4)	0.0149 (8)	
C5	0.3787 (8)	0.5164 (2)	0.7368 (4)	0.0168 (8)	
H5	0.3330	0.4866	0.6559	0.020*	
C6	0.5907 (9)	0.4956 (2)	0.8401 (4)	0.0186 (9)	
H6	0.6911	0.4508	0.8296	0.022*	
C7	0.6596 (9)	0.5392 (2)	0.9593 (4)	0.0182 (8)	
H7	0.8068	0.5238	1.0282	0.022*	
C8	0.5155 (9)	0.6048 (2)	0.9785 (5)	0.0180 (8)	
H8	0.5608	0.6346	1.0596	0.022*	
C8A	0.3030 (8)	0.6250 (2)	0.8747 (4)	0.0146 (8)	
S9	0.0967 (2)	0.70586 (5)	0.87325 (11)	0.0174 (2)	
C9A	−0.0728 (8)	0.6813 (2)	0.7133 (4)	0.0163 (8)	
C10	−0.0297 (8)	0.5298 (2)	0.4485 (4)	0.0158 (8)	
C11	0.1148 (8)	0.5411 (2)	0.3255 (4)	0.0172 (8)	
H11	0.1567	0.5896	0.2972	0.021*	
C12	0.1981 (9)	0.4824 (2)	0.2441 (4)	0.0182 (8)	
H12	0.3004	0.4908	0.1622	0.022*	
C13	0.1307 (8)	0.4114 (2)	0.2833 (4)	0.0156 (8)	
C14	−0.0266 (9)	0.3998 (2)	0.4026 (5)	0.0205 (9)	
H14	−0.0819	0.3516	0.4270	0.025*	
C15	−0.1006 (8)	0.4583 (2)	0.4841 (4)	0.0192 (9)	
H15	−0.2019	0.4498	0.5663	0.023*	
O1	0.2001 (6)	0.35027 (15)	0.2094 (3)	0.0199 (6)	
C16	0.3593 (9)	0.3608 (2)	0.0861 (4)	0.0198 (9)	
H16A	0.3901	0.3134	0.0411	0.030*	
H16B	0.5466	0.3833	0.1145	0.030*	
H16C	0.2497	0.3930	0.0188	0.030*	
S1	0.2806 (2)	0.78392 (5)	0.26484 (11)	0.0180 (2)	
O2	0.3759 (7)	0.7346 (2)	0.1596 (3)	0.0333 (8)	
O3	0.2090 (6)	0.74833 (15)	0.3937 (3)	0.0228 (6)	
O4	0.0395 (6)	0.83188 (17)	0.2081 (3)	0.0256 (7)	
O5	0.5298 (6)	0.83793 (17)	0.3069 (4)	0.0277 (7)	
H5O	0.6634	0.8370	0.2413	0.042*	

### Atomic displacement parameters (Å^2^)

**Table:**

	*U*^11^	*U*^22^	*U*^33^	*U*^12^	*U*^13^	*U*^23^
Se1	0.01455 (16)	0.01458 (16)	0.02001 (18)	0.00098 (18)	0.00359 (13)	0.00010 (19)
C2	0.0167 (19)	0.018 (2)	0.018 (2)	−0.0019 (16)	0.0052 (16)	0.0018 (16)
C3	0.0159 (19)	0.0149 (18)	0.019 (2)	−0.0012 (15)	0.0058 (16)	0.0005 (16)
N4	0.0130 (15)	0.0124 (15)	0.0192 (17)	−0.0010 (13)	0.0049 (13)	−0.0009 (13)
C4A	0.0115 (18)	0.0145 (18)	0.019 (2)	−0.0025 (15)	0.0036 (15)	−0.0001 (15)
C5	0.017 (2)	0.0164 (19)	0.018 (2)	−0.0027 (16)	0.0047 (16)	−0.0004 (16)
C6	0.0153 (19)	0.0165 (19)	0.025 (2)	0.0015 (16)	0.0085 (17)	0.0024 (17)
C7	0.0135 (19)	0.022 (2)	0.019 (2)	0.0007 (16)	0.0020 (16)	0.0031 (17)
C8	0.016 (2)	0.0172 (19)	0.021 (2)	−0.0006 (16)	0.0050 (17)	0.0012 (17)
C8A	0.0156 (19)	0.0124 (18)	0.016 (2)	0.0009 (15)	0.0053 (15)	−0.0010 (15)
S9	0.0163 (4)	0.0168 (5)	0.0192 (5)	0.0003 (3)	0.0028 (4)	−0.0013 (4)
C9A	0.0155 (19)	0.0165 (18)	0.0175 (19)	−0.0055 (16)	0.0054 (15)	−0.0042 (16)
C10	0.0132 (18)	0.0137 (18)	0.021 (2)	−0.0009 (15)	0.0019 (16)	−0.0011 (16)
C11	0.0149 (19)	0.0165 (19)	0.020 (2)	−0.0005 (16)	0.0007 (16)	0.0008 (16)
C12	0.018 (2)	0.019 (2)	0.018 (2)	−0.0002 (16)	0.0034 (16)	0.0003 (16)
C13	0.0148 (19)	0.0155 (19)	0.016 (2)	0.0012 (15)	0.0000 (15)	−0.0035 (16)
C14	0.019 (2)	0.018 (2)	0.025 (2)	−0.0031 (17)	0.0067 (17)	0.0013 (17)
C15	0.0171 (19)	0.021 (2)	0.021 (2)	−0.0022 (16)	0.0055 (17)	−0.0016 (17)
O1	0.0223 (15)	0.0163 (14)	0.0223 (15)	−0.0003 (12)	0.0096 (13)	−0.0003 (12)
C16	0.021 (2)	0.021 (2)	0.018 (2)	−0.0003 (17)	0.0067 (17)	−0.0029 (17)
S1	0.0164 (5)	0.0182 (5)	0.0197 (5)	0.0014 (4)	0.0029 (4)	0.0002 (4)
O2	0.0372 (17)	0.036 (2)	0.0270 (14)	0.0050 (17)	0.0053 (13)	−0.0125 (16)
O3	0.0204 (15)	0.0232 (15)	0.0252 (15)	0.0021 (12)	0.0041 (12)	0.0073 (12)
O4	0.0152 (15)	0.0258 (16)	0.0353 (18)	0.0019 (12)	−0.0007 (13)	0.0102 (14)
O5	0.0167 (15)	0.0263 (17)	0.0406 (18)	−0.0014 (13)	0.0064 (14)	−0.0019 (15)

### Geometric parameters (Å, º)

**Table:**

Se1—C9A	1.834 (4)	C10—C15	1.394 (5)
Se1—C2	1.859 (4)	C10—C11	1.399 (6)
C2—C3	1.345 (5)	C11—C12	1.391 (6)
C2—H2	0.9500	C11—H11	0.9500
C3—N4	1.415 (5)	C12—C13	1.390 (6)
C3—C10	1.485 (5)	C12—H12	0.9500
N4—C9A	1.373 (5)	C13—O1	1.367 (5)
N4—C4A	1.412 (5)	C13—C14	1.404 (6)
C4A—C5	1.385 (5)	C14—C15	1.376 (6)
C4A—C8A	1.397 (5)	C14—H14	0.9500
C5—C6	1.386 (6)	C15—H15	0.9500
C5—H5	0.9500	O1—C16	1.439 (5)
C6—C7	1.397 (6)	C16—H16A	0.9800
C6—H6	0.9500	C16—H16B	0.9800
C7—C8	1.391 (6)	C16—H16C	0.9800
C7—H7	0.9500	S1—O2	1.436 (3)
C8—C8A	1.388 (6)	S1—O3	1.441 (3)
C8—H8	0.9500	S1—O4	1.489 (3)
C8A—S9	1.759 (4)	S1—O5	1.549 (3)
S9—C9A	1.710 (4)	O5—H5O	0.9086
			
C9A—Se1—C2	84.92 (18)	C15—C10—C11	118.3 (4)
C3—C2—Se1	114.8 (3)	C15—C10—C3	123.9 (4)
C3—C2—H2	122.6	C11—C10—C3	117.7 (3)
Se1—C2—H2	122.6	C12—C11—C10	121.1 (4)
C2—C3—N4	112.5 (4)	C12—C11—H11	119.5
C2—C3—C10	123.7 (4)	C10—C11—H11	119.5
N4—C3—C10	123.7 (3)	C11—C12—C13	119.6 (4)
C9A—N4—C4A	113.2 (3)	C11—C12—H12	120.2
C9A—N4—C3	114.2 (3)	C13—C12—H12	120.2
C4A—N4—C3	132.6 (3)	O1—C13—C12	124.0 (4)
C5—C4A—C8A	120.3 (4)	O1—C13—C14	116.3 (4)
C5—C4A—N4	128.7 (4)	C12—C13—C14	119.7 (4)
C8A—C4A—N4	111.0 (3)	C15—C14—C13	119.9 (4)
C4A—C5—C6	118.3 (4)	C15—C14—H14	120.0
C4A—C5—H5	120.9	C13—C14—H14	120.0
C6—C5—H5	120.9	C14—C15—C10	121.3 (4)
C5—C6—C7	121.3 (4)	C14—C15—H15	119.4
C5—C6—H6	119.4	C10—C15—H15	119.4
C7—C6—H6	119.4	C13—O1—C16	117.3 (3)
C8—C7—C6	120.9 (4)	O1—C16—H16A	109.5
C8—C7—H7	119.6	O1—C16—H16B	109.5
C6—C7—H7	119.6	H16A—C16—H16B	109.5
C8A—C8—C7	117.4 (4)	O1—C16—H16C	109.5
C8A—C8—H8	121.3	H16A—C16—H16C	109.5
C7—C8—H8	121.3	H16B—C16—H16C	109.5
C8—C8A—C4A	121.9 (4)	O2—S1—O3	113.9 (2)
C8—C8A—S9	125.9 (3)	O2—S1—O4	112.44 (19)
C4A—C8A—S9	112.2 (3)	O3—S1—O4	110.80 (18)
C9A—S9—C8A	90.01 (19)	O2—S1—O5	108.31 (19)
N4—C9A—S9	113.6 (3)	O3—S1—O5	106.59 (18)
N4—C9A—Se1	113.5 (3)	O4—S1—O5	104.13 (18)
S9—C9A—Se1	132.9 (2)	S1—O5—H5O	110.3
			
C9A—Se1—C2—C3	0.2 (3)	C4A—N4—C9A—S9	0.3 (4)
Se1—C2—C3—N4	0.6 (4)	C3—N4—C9A—S9	−178.3 (3)
Se1—C2—C3—C10	−175.7 (3)	C4A—N4—C9A—Se1	−180.0 (3)
C2—C3—N4—C9A	−1.3 (5)	C3—N4—C9A—Se1	1.5 (4)
C10—C3—N4—C9A	174.9 (4)	C8A—S9—C9A—N4	0.1 (3)
C2—C3—N4—C4A	−179.5 (4)	C8A—S9—C9A—Se1	−179.6 (3)
C10—C3—N4—C4A	−3.3 (6)	C2—Se1—C9A—N4	−0.9 (3)
C9A—N4—C4A—C5	−179.1 (4)	C2—Se1—C9A—S9	178.8 (3)
C3—N4—C4A—C5	−0.9 (7)	C2—C3—C10—C15	−110.1 (5)
C9A—N4—C4A—C8A	−0.6 (4)	N4—C3—C10—C15	74.1 (5)
C3—N4—C4A—C8A	177.6 (4)	C2—C3—C10—C11	66.4 (5)
C8A—C4A—C5—C6	−0.5 (6)	N4—C3—C10—C11	−109.4 (4)
N4—C4A—C5—C6	177.9 (4)	C15—C10—C11—C12	−2.9 (6)
C4A—C5—C6—C7	−0.1 (6)	C3—C10—C11—C12	−179.6 (4)
C5—C6—C7—C8	0.4 (6)	C10—C11—C12—C13	1.6 (6)
C6—C7—C8—C8A	−0.2 (6)	C11—C12—C13—O1	179.2 (4)
C7—C8—C8A—C4A	−0.3 (6)	C11—C12—C13—C14	1.5 (6)
C7—C8—C8A—S9	−178.7 (3)	O1—C13—C14—C15	178.9 (4)
C5—C4A—C8A—C8	0.7 (6)	C12—C13—C14—C15	−3.3 (6)
N4—C4A—C8A—C8	−177.9 (4)	C13—C14—C15—C10	1.9 (6)
C5—C4A—C8A—S9	179.3 (3)	C11—C10—C15—C14	1.2 (6)
N4—C4A—C8A—S9	0.7 (4)	C3—C10—C15—C14	177.7 (4)
C8—C8A—S9—C9A	178.1 (4)	C12—C13—O1—C16	1.7 (5)
C4A—C8A—S9—C9A	−0.4 (3)	C14—C13—O1—C16	179.5 (3)

### Hydrogen-bond geometry (Å, º)

**Table:**

*D*—H···*A*	*D*—H	H···*A*	*D*···*A*	*D*—H···*A*
O5—H5*O*···O4^i^	0.91	1.80	2.610 (4)	147
C6—H6···O4^ii^	0.95	2.54	3.494 (5)	178
C8—H8···O2^iii^	0.95	2.26	3.022 (5)	137

Symmetry codes: (i) *x*+1, *y*, *z*; (ii) −*x*+1, *y*−1/2, −*z*+1; (iii) *x*, *y*, *z*+1.
